# Supplementary material for: Itaconate ameliorates autoimmunity by modulating T cell imbalance via metabolic and epigenetic reprogramming
Source: Nat Commun. 2023 Feb 27;14:984. doi: 10.1038/s41467-023-36594-x (PMC9970976; doi:10.1038/s41467-023-36594-x)
Supplement: Supplementary file 1 — Supplementary Information [file 41467_2023_36594_MOESM1_ESM.pdf]

Supplemental Information

Itaconate ameliorates autoimmunity by modulating T cell imbalance via metabolic and epigenetic reprogramming

Kuniyuki Aso, Michihito Kono, Masatoshi Kanda, Yuki Kudo, Kodai Sakiyama, Ryo Hisada, Kohei Karino, Yusho Ueda, Daigo Nakazawa, Yuichiro Fujieda, Masaru Kato, Olga Amengual and Tatsuya Atsumi

This file includes: Supplementary Fig.1 to 6

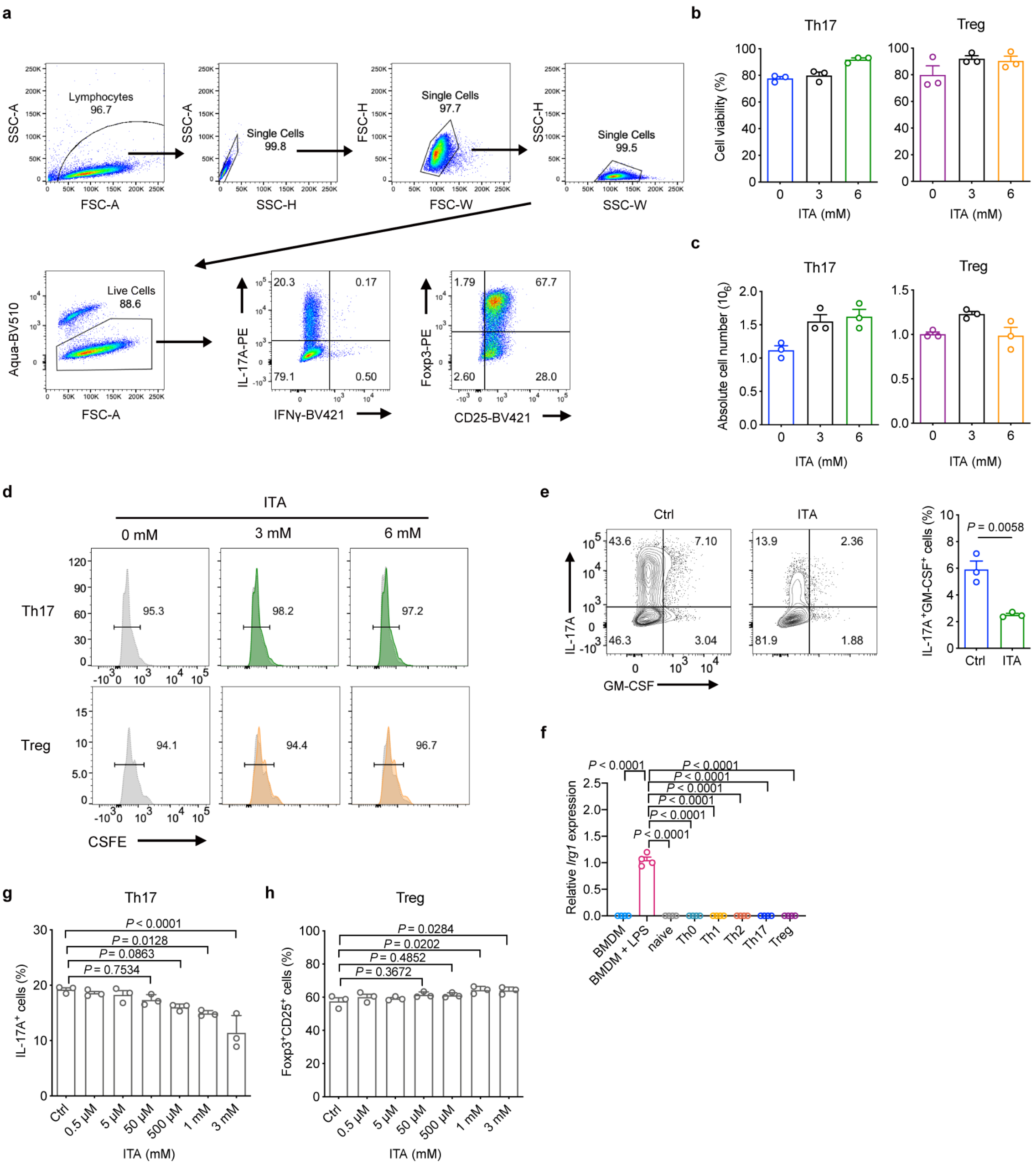

Supplementary Fig. 1 Itaconate modulates Th17 and Treg differentiation without affecting cellular viability and proliferation. a Gating strategy for the flow cytometric analyses of in vitro

Th17- and Treg-polarizing T cells. **b, c** Cell viability (**b**) and absolute cell number (**c**) in ITA-treated Th17- and Treg-polarizing T cells at the end of the 3-day culture (n = 3, each condition). **d** Representative flow cytometry plot displaying T cell proliferation assessed as CSFE dilution in the presence or absence of ITA (0, 3, and 6 mM) after 3-day culture. **e** Flow cytometric analyses of differentiation of Th17 with ITA under pathogenic conditions (with IL-6, IL-23, and IL-1β) (n = 3). **f** Quantitative PCR analysis of *Irg1* in naive $CD4^+$ T, Th0, Th1, Th2, Th17, Treg cells, and bone-marrow-derived macrophages (BMDM) in the presence or absence of LPS (n = 4, each condition). **g, h** Cumulative data of the differentiation of murine naive $CD4^+$ T cells from wild-type B6 mice activated under Th17 (**g**), and Treg (**h**) cell conditions treated with indicated ITA concentrations after 3 days of culture (n = 3, each condition). P values are calculated using two-way ANOVA for (**b, c, f–h**) and two-tailed unpaired Student's t-test for (**e**). Data are representative of mean ± s.e.m. Source data are provided as a Source Data file.

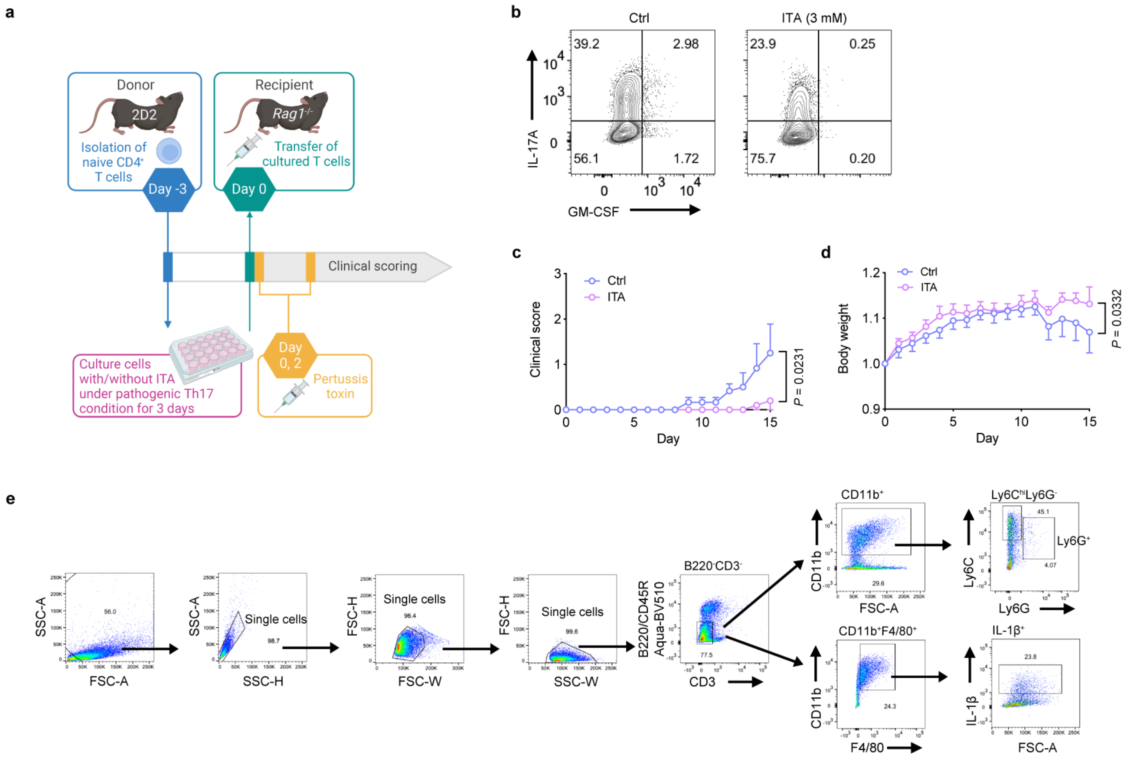

Supplementary Fig. 2 Effect of itaconate in adoptive transfer experimental autoimmune encephalomyelitis model. a Schematic of adoptive transfer experimental autoimmune encephalomyelitis (tEAE) model. Created with BioRender.com. **b** Naive CD4^{+} T cells from 2D2 mice were differentiated under pathogenic Th17 conditions with or without itaconate (ITA, 3 mM) in vitro. The expression of IL-17A and GM-CSF was determined by flow cytometry. Representative plots are shown. **c, d** *Rag1*-deficient mice were treated with 50 mg/kg of itaconate intraperitoneal injection every other day from day 0 to day 14 following the adoptive transfer of

pathogenic Th17-polarizing $CD4^{+}$ T cells from 2D2 mice. Clinical scores (**c**) and body weight (**d**) in the mice treated with PBS (Ctrl, n = 6) or ITA (n = 5). **e** Gating strategy for the flow cytometric analyses of macrophages, $Ly6C^{hi}$ monocytes, and neutrophils in the spinal cords 14 days after the induction of tEAE. Data of **b** are representative of three independent experiments with similar results. P values are calculated using two-way ANOVA for (**c**, **d**). Data are representative of mean ± s.e.m. Source data are provided as a Source Data file.

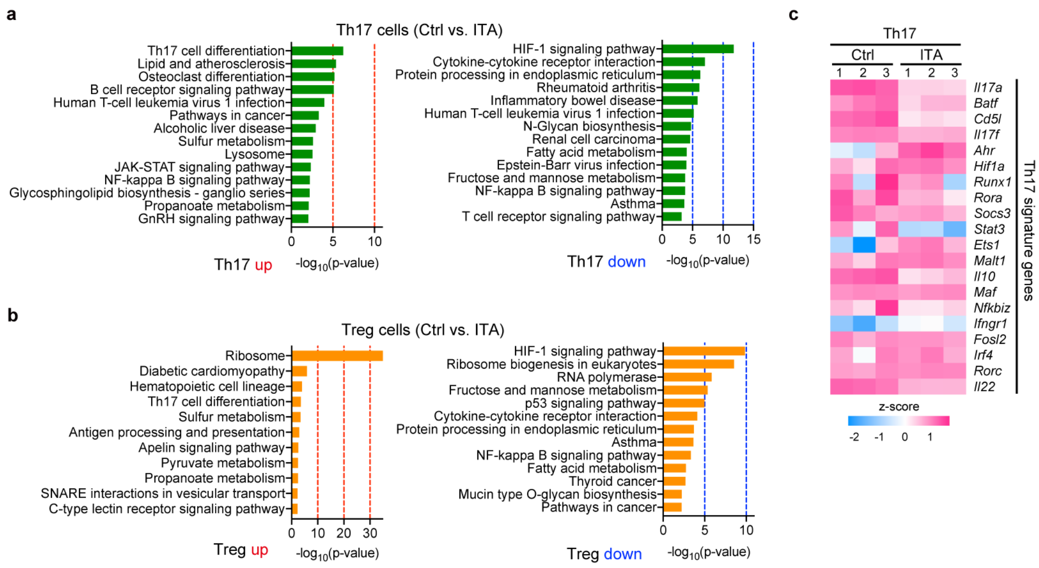

Supplementary Fig. 3 Effect of itaconate on gene expression in Th17- and Treg-polarizing T cells. a–c Th17- and Treg-polarizing T cells from B6 mice in the presence or absence of ITA after 2 days of culture were subjected to RNA sequencing (n = 3 in each condition, independent experiments). Kyoto Encyclopedia of Genes and Genomes (KEGG) pathway analysis of differentially expressed genes from ITA treated Th17- (**a**) and Treg- (**b**) polarizing T cells compared to that of control (Ctrl). P-value for (**a, b**) indicates gene enrichment analysis test implemented in Metascape without adjustment for multiple comparisons. Heatmap (**c**) shows relative expression (z-score) of Th17 signature genes in Th17 condition according to RNA-seq. Source data are provided as a Source Data file.

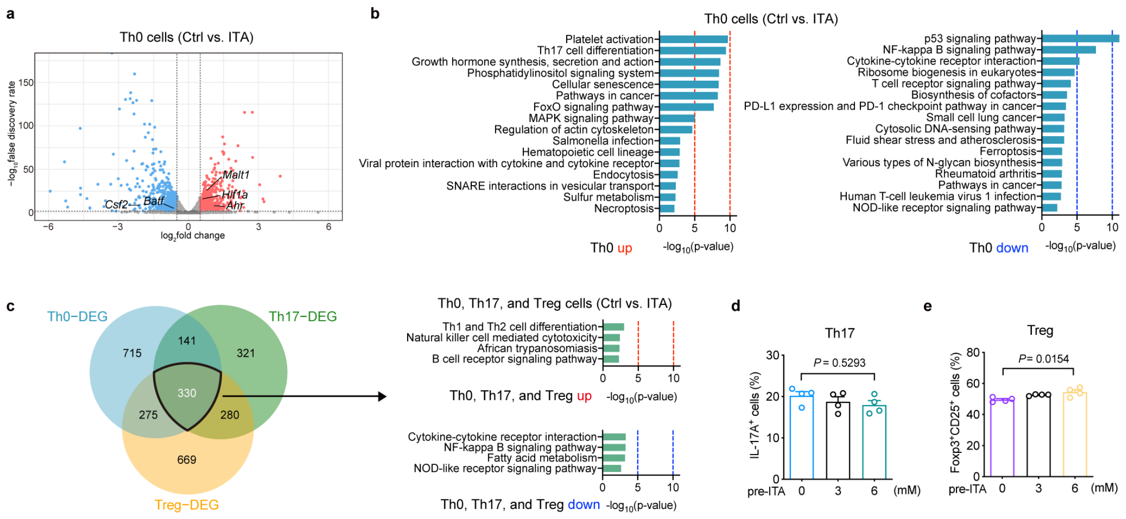

Supplementary Fig. 4 Influence of itaconate on activated naive CD4⁺ T cells in the absence of unique cytokine signals for Th17 or Treg cells. a–c For the Th0 condition, murine naive CD4$^+$ T cells from B6 mice were stimulated with CD3/CD28 antibodies in the presence or absence of ITA (6 mM) and subjected to RNA-seq after 2 days (n = 3 in each condition, independent experiments). Volcano plot of differential gene expression in ITA-treated Th0 cells as compared to control (Ctrl) (log2 fold-change > 0.5 and adjusted P-value < 0.01) (**a**). KEGG pathway analysis of differentially expressed genes from ITA-treated Th0 cells compared to Ctrl (**b**). Venn diagram displaying the overlap between differentially expressed genes between ITA-treated and control T cells under Th0, Th17, and Treg conditions (**c**). P-value for (**b, c**) indicates gene enrichment analysis test implemented in Metascape without adjustment for multiple comparisons. **d, e** Murine

naive CD4$^+$ T cells from B6 mice were stimulated with CD3/CD28 antibodies in the presence or absence of ITA (6 mM) for 1 day. The cells were washed and cultured under Th17 (**d**) and Treg (**e**) conditions for an additional 2 days. On day 3, the cells were collected and analyzed using flow cytometry (n = 4, each condition). P values are calculated using one-way ANOVA with Bonferroni post hoc test for (**d, e**). Data are representative of mean ± s.e.m. Source data are provided as a Source Data file.

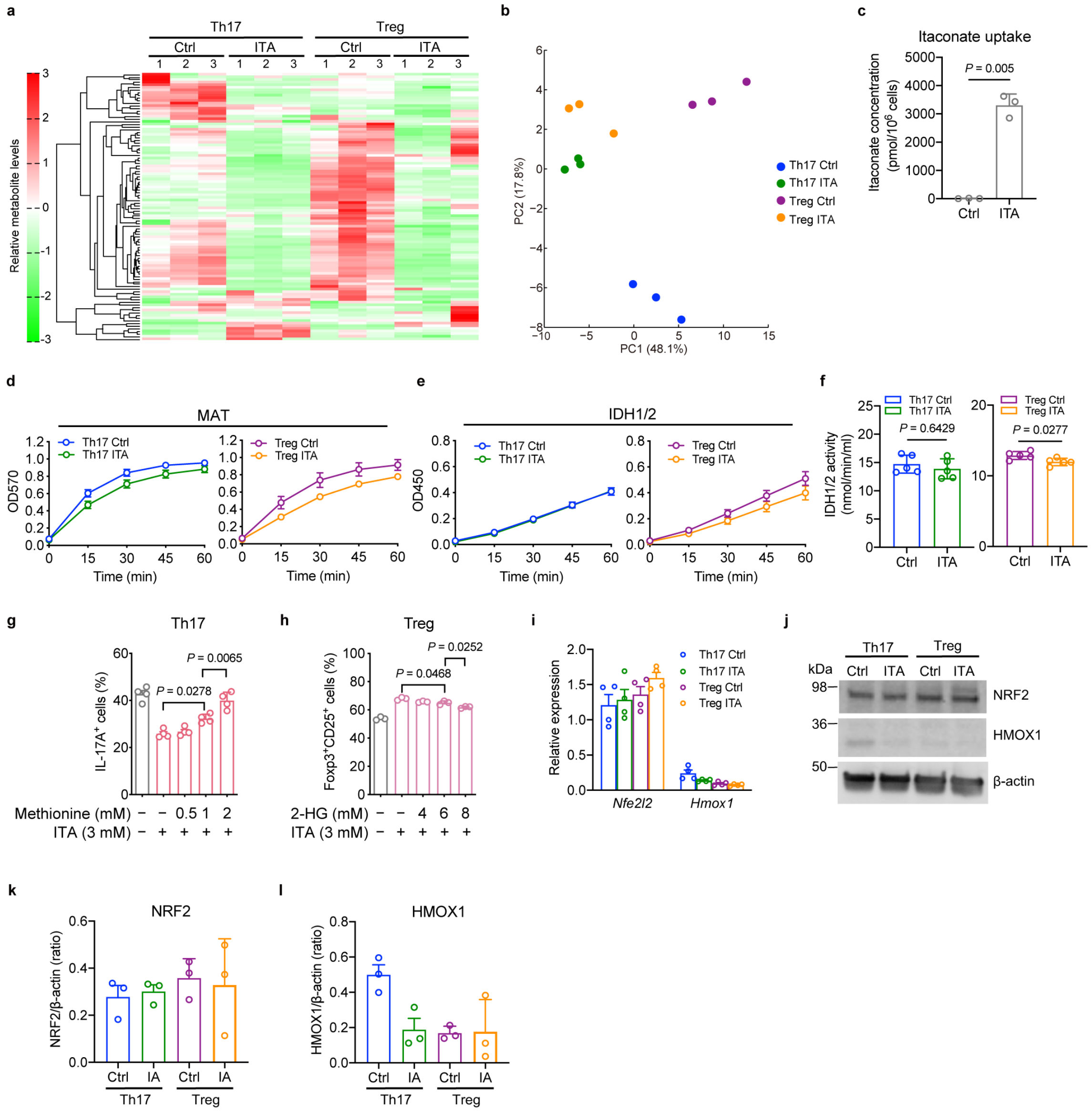

Supplementary Fig. 5 Effect of Itaconate on metabolite levels, enzymatic activity, and transcription factors in Th17- and Treg-polarizing T cells. a,**b** Heatmap (**a**) and principal component analysis (**b**) of global metabolites from Th17- and Treg-polarizing T cells with or

without ITA as measured by capillary electrophoresis time-of-flight mass spectrometry. **c** Intracellular levels of ITA in activated naïve CD4$^+$ T cells after CD3/CD28 stimulation in the presence or absence of ITA (n = 3). **d**,**e** Enzymatic activity of methionine adenosyltransferase (MAT) (**d**) (n = 3, each condition) and isocitrate dehydrogenase (IDH)-1 and IDH-2 (**e**) (n = 4, each condition) in Th17- and Treg-polarizing T cells with or without ITA. Real-time OD values indicating the catalytic reaction. **f** IDH1/2 activity of the whole-cell extract from Th17- and Treg-polarizing T cells in the presence or absence of itaconate (n = 5, each condition). **g, h** Cumulative data of the differentiation of murine naive CD4$^+$ T cells from wild-type B6 mice activated under Th17 (**g**), and Treg (**h**) cell conditions treated with or without ITA (3 mM) and/or various concentrations of methionine (**g**) or 2-HG (**h**) after 3 days of culture (methionine, n = 4; 2-HG, n = 3). **i** Quantitative PCR analysis of *Nfe2l2,* and *Hmox1* in Th17 and Treg cells (n = 4). **j–l** Representative (**j**) and cumulative (**k, l**) data of the expression of mouse NRF2 and HMOX1 in Th17 and Treg cells with or without ITA (n = 3). P values are calculated using two-tailed unpaired Student's t-test for (**c**, **f**) and one-way ANOVA with Bonferroni post hoc test for (**g–i, k, l**). Data are representative of mean ± s.e.m. Source data are provided as a Source Data file.

Supplementary Fig.5j

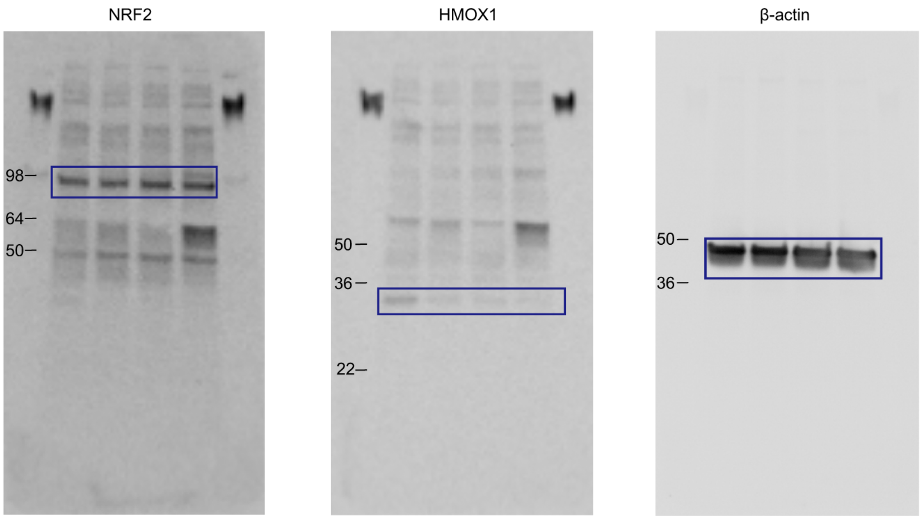

Supplementary Fig. 6 Full scans for Fig. 5j.
