# Supplementary material for: Itaconate ameliorates autoimmunity by modulating T cell imbalance via metabolic and epigenetic reprogramming
Source: Nat Commun. 2023 Feb 27;14:984. doi: 10.1038/s41467-023-36594-x (PMC9970976; doi:10.1038/s41467-023-36594-x)
Supplement: Supplementary file 3 — Reporting Summary [file 41467_2023_36594_MOESM3_ESM.pdf]

nature portfolio

Corresponding author(s): Michihito Kono

Last updated by author(s): Jan 13, 2023

Reporting Summary

Nature Portfolio wishes to improve the reproducibility of the work that we publish. This form provides structure for consistency and transparency in reporting. For further information on Nature Portfolio policies, see our Editorial Policies and the Editorial Policy Checklist.

Statistics

For all statistical analyses, confirm that the following items are present in the figure legend, table legend, main text, or Methods section.

n/a	Confirmed	
☐	☒	The exact sample size (*n*) for each experimental group/condition, given as a discrete number and unit of measurement
☐	☒	A statement on whether measurements were taken from distinct samples or whether the same sample was measured repeatedly
☐	☒	The statistical test(s) used AND whether they are one- or two-sided<br>*Only common tests should be described solely by name; describe more complex techniques in the Methods section.*
☒	☐	A description of all covariates tested
☒	☐	A description of any assumptions or corrections, such as tests of normality and adjustment for multiple comparisons
☐	☒	A full description of the statistical parameters including central tendency (e.g. means) or other basic estimates (e.g. regression coefficient) AND variation (e.g. standard deviation) or associated estimates of uncertainty (e.g. confidence intervals)
☐	☒	For null hypothesis testing, the test statistic (e.g. *F*, *t*, *r*) with confidence intervals, effect sizes, degrees of freedom and *P* value noted<br>*Give P values as exact values whenever suitable.*
☒	☐	For Bayesian analysis, information on the choice of priors and Markov chain Monte Carlo settings
☒	☐	For hierarchical and complex designs, identification of the appropriate level for tests and full reporting of outcomes
☒	☐	Estimates of effect sizes (e.g. Cohen's *d*, Pearson's *r*), indicating how they were calculated

Our web collection on statistics for biologists contains articles on many of the points above.

Software and code

Policy information about availability of computer code

Data collection	FACS Aria III (BD) was used for flow cytometry data collection.<br>RNA-seq were sequenced by Illumina NovaSeq (Illumina).<br>ATAC-seq data were sequenced by NextSeq 500 sequencer (Illumina).
Data analysis	FlowJo v10.6.1 (BD) was used to analyze flow cytometry data. GraphPad Prism v8.4.3 (GraphPad Software) was used for graphs and statistical analysis. R platform v3.6.1, DESeq2 v1.24.0, STAR v2.7.1a, and Metascape v3.5 web-based platform were used for analysis of RNA-seq data. Seahorse Wave v2.6.0 (Agilent technologies) was used for metabolic studies. Keyence BZ-X Analyzer software (Keyence) were used for analysis of spinal specimens.

For manuscripts utilizing custom algorithms or software that are central to the research but not yet described in published literature, software must be made available to editors and reviewers. We strongly encourage code deposition in a community repository (e.g. GitHub). See the Nature Portfolio guidelines for submitting code & software for further information.

Data

Policy information about availability of data

All manuscripts must include a data availability statement. This statement should provide the following information, where applicable:

- Accession codes, unique identifiers, or web links for publicly available datasets
- A description of any restrictions on data availability
- For clinical datasets or third party data, please ensure that the statement adheres to our policy

The RNA-seq data used for Figure 3a-h are publicly available at the NCBI Gene Expression Omnibus (GEO) under the accession number GSE182895 [https://www.ncbi.nlm.nih.gov/geo/query/acc.cgi?acc=GSE182895]. ATAC-seq data used for Figure b-f are publicly available at the NCBI GEO under the accession number GSE207941 [https://www.ncbi.nlm.nih.gov/geo/query/acc.cgi?acc=GSE207941]. The other data are available in the main text or the supplementary information and they would be available upon reasonable request to the corresponding author. KEEG pathway analysis in the manuscript indicates the integrated analysis in Metascape. Please find the references to the parent algorithms (Metascape).

Human research participants

Policy information about studies involving human research participants and Sex and Gender in Research.

Reporting on sex and gender	Not applicable
Population characteristics	Not applicable
Recruitment	Not applicable
Ethics oversight	Not applicable

Note that full information on the approval of the study protocol must also be provided in the manuscript.

Field-specific reporting

Please select the one below that is the best fit for your research. If you are not sure, read the appropriate sections before making your selection.

☒ Life sciences ☐ Behavioural & social sciences ☐ Ecological, evolutionary & environmental sciences

For a reference copy of the document with all sections, see nature.com/documents/nr-reporting-summary-flat.pdf

Life sciences study design

All studies must disclose on these points even when the disclosure is negative.

Sample size	No statistical methods were used to predetermine sample sizes. Sample sizes were based on pilot experiments conducted in the same laboratory and comparable to similar studies in the field. The precise number of animals were indicated in the figure legend.
Data exclusions	No data were excluded from analyses.
Replication	We repeated each experiment at least twice to ensure consistent results. All repeats showed similar trends.
Randomization	Mice used in the in vitro and vivo testing were randomly assigned to experimental groups.
Blinding	The evaluation and scoring of histopathology of HE-stained tissue sections was performed in a blinded fashion.<br>In the other experiments, no blinding was used during allocation of experimental groups, because all data collection and analysis is quantitative and not qualitative in nature. To avoid introducing bias, samples were measured in a standardized way.

Reporting for specific materials, systems and methods

We require information from authors about some types of materials, experimental systems and methods used in many studies. Here, indicate whether each material, system or method listed is relevant to your study. If you are not sure if a list item applies to your research, read the appropriate section before selecting a response.

Materials & experimental systems

n/a	Involved in the study
☐	☒ Antibodies
☒	☐ Eukaryotic cell lines
☒	☐ Palaeontology and archaeology
☐	☒ Animals and other organisms
☒	☐ Clinical data
☒	☐ Dual use research of concern

Methods

n/a	Involved in the study
☒	☐ ChIP-seq
☐	☒ Flow cytometry
☒	☐ MRI-based neuroimaging

Antibodies

Antibodies used

Antibodies for flowcytometry:
anti-IL-17A (PE) (TC11-18H10.1; #506904; Biolegend; 1:50)
anti-IFN-γ (BV421) (XMG1.2; #505830; Biolegend; 1:50)
anti-Foxp3 (PE) (FJK-16s; #12-5773-82; eBioscience; 1:50)
anti-IL-4 (PE) (11B11; #504104; Biolegend; 1:50)
anti-CD25 (BV421) (3C7; #564370; BD; 1:100)
anti-RORγt (APC) (B2D; #17-6981-80; eBioscience; 1:50)
anti-GM-CSF (APC) (MP1-22E9; #17-7331-82; eBioscience; 1:50)
anti-HIF-1α (APC) (241812; #IC1935A; R&D Systems; 1:50)
anti-CD4 (FITC) (H129.19; #130308; Biolegend; 1:100)
anti-CD90.2 (PE-Cy7) (53-2.1; #140310; Biolegend; 1:100)
anti-CD16/CD32 mix (2.4G2, #553141, BD Biosciences; 0.01 mg/mL)
anti-CD3 (PerCP-Cy5.5) (17A2, #100217, Biolegend; 1:100)
anti-CD11b (APC-Cy7) (M1/70, #557657, Biolegend; 1:100)
anti-Ly6C (FITC) (HK1.4, #128006, Biolegend; 1:100)
anti-Ly6G (APC) (1A8, #127613, Biolegend; 1:100)
anti-B220/CD45R (BV510) (RA3-6B2, #563103, Biolegend; 1:100)
anti-F4/80 (PE-Cy7) (BM8, #123113, Biolegend; 1:100)
anti-pro-IL-1β (PE) (NJTEN3, #12-7114-80, eBioscience; 1:50)

Antibodies for ChIP-qPCR:
Rat monoclonal anti-Mouse RORγt (AFKJS-9; #14-6988-82; eBioscience; 40 ug/ml)
Rat monoclonal IgG2a kappa Isotype Control (eBR2a; #14-4321-82; eBioscience; 40 ug/ml)

Antibodies for Immunoblotting:
Rabbit monoclonal anti-mouse NRF2 (D1Z9C, #12721S, Cell Signaling Technology; 1:500)
Rabbit monoclonal anti-mouse HO-1 (E6Z5G, #82206, Cell Signaling Technology; 1:500)
Mouse monoclonal anti-β-actin (AC-15, #A3854, Sigma-Aldrich; 1:50000)
Goat anti-rabbit IgG (whole molecule) peroxidase-conjugated (#A0545, Sigma-Aldrich; 1:80000)
Rabbit anti-mouse IgG (whole molecule) peroxidase-conjugated (#A9044, Sigma-Aldrich; 1:120000)

Validation

All antibodies were verified by manufacturers and widely used in the published literatures. A full reference list can be found on the official website of the manufacturer.
anti-IL-17A (PE) https://www.biolegend.com/ja-jp/products/pe-anti-mouse-il-17a-antibody-1633?GroupID=GROUP24
anti-IFN-γ (BV421) https://www.biolegend.com/ja-jp/products/brilliant-violet-421-anti-mouse-ifn-gamma-antibody-7154
anti-Foxp3 (PE) https://www.thermofisher.com/antibody/product/FOXP3-Antibody-clone-FJK-16s-Monoclonal/12-5773-82
anti-IL-4 (PE) https://www.biolegend.com/ja-jp/products/pe-anti-mouse-il-4-antibody-893
anti-CD25 (BV421) https://www.bdbiosciences.com/tw/applications/research/t-cell-immunology/regulatory-t-cells/surface-markers/mouse/bv421-rat-anti-mouse-cd25-3c7/p/564370
anti-RORγt (APC) https://www.thermofisher.com/antibody/product/ROR-gamma-t-Antibody-clone-B2D-Monoclonal/17-6981-82
anti-GM-CSF (APC) https://www.thermofisher.com/antibody/product/GM-CSF-Antibody-clone-MP1-22E9-Monoclonal/17-7331-82
anti-HIF-1α (APC) https://www.rndsystems.com/products/human-mouse-hif-1-alpha-hif1a-apc-conjugated-antibody-241812_ic1935a
anti-CD4 (FITC) https://www.biolegend.com/ja-jp/products/fitc-anti-mouse-cd4-antibody-5487?GroupID=BLG4211
anti-CD90.2 (PE-Cy7) https://www.biolegend.com/ja-jp/neuroscience-1/pe-cyanine7-anti-mouse-cd90-2-thy-1-2-antibody-6868?GroupID=BLG8878
anti-CD16/CD32 mix https://www.bdbiosciences.com/en-au/products/reagents/flow-cytometry-reagents/research-reagents/single-color-antibodies-ruo/purified-rat-anti-mouse-cd16-cd32-mouse-bd-fc-block.553141
anti-CD3 (PerCP-Cy5.5) https://www.biolegend.com/ja-jp/products/percp-cyanine5-5-anti-mouse-cd3-antibody-5596
anti-CD11b (APC-Cy7) https://www.biolegend.com/ja-jp/products/apc-cyanine7-anti-mouse-human-cd11b-antibody-3930?GroupID=BLG10616
anti-Ly6C (FITC) https://www.biolegend.com/ja-jp/products/fitc-anti-mouse-ly-6c-antibody-4896
anti-Ly6G (APC) https://www.biolegend.com/ja-jp/products/apc-anti-mouse-ly-6g-antibody-6115
anti-B220/CD45R (BV510) https://www.biolegend.com/ja-jp/products/brilliant-violet-510-anti-mouse-human-cd45r-b220-antibody-7996
anti-F4/80 (PE-Cy7) https://www.biolegend.com/ja-jp/products/pe-cyanine7-anti-mouse-f4-80-antibody-4070?GroupID=BLG5319
anti-pro-IL-1β (PE) https://www.thermofisher.com/antibody/product/IL-1-beta-Pro-form-Antibody-clone-NJTEN3-Monoclonal/12-7114-82

Rat monoclonal anti-Mouse RORγt https://www.thermofisher.com/antibody/product/ROR-gamma-t-Antibody-clone-AFKJS-9-

Monoclonal/14-6988-82
Rat monoclonal IgG2a kappa Isotype Control https://www.thermofisher.com/antibody/product/Rat-IgG2a-kappa-clone-eBR2a-Isotype-Control/14-4321-82

Rabbit monoclonal anti-mouse NRF2 https://www.cellsignal.jp/products/primary-antibodies/nrf2-d1z9c-xp-rabbit-mab/12721
Rabbit monoclonal anti-mouse HO-1 https://en.cellsignal.jp/products/primary-antibodies/ho-1-e6z5g-rabbit-mab/82206
Mouse monoclonal anti-β-actin https://www.sigmaaldrich.com/JP/ja/product/sigma/a3854

Animals and other research organisms

Policy information about studies involving animals; ARRIVE guidelines recommended for reporting animal research, and Sex and Gender in Research

Laboratory animals	All mice were bred in house and maintained in temperature- and humidity-controlled facilities under pathogen-free conditions at the Hokkaido University (Japan) under approved protocols, group housed with free access to food and water and 12h light/dark cycles. C57BL/6J mice were purchased from Charles River Laboratories (Wilmington, MA). 2D2 (C57BL/6-Tg (Tcra2D2, Tcrb2D2) 1Kuch/J) and Rag1−/− (B6.129S7-Rag1tm1Mom/Jmice) were purchased from Jackson Laboratories (Bar Harbor, ME). Nrf2−/− (B6.129X1-Nfe2l2tm1Ywk/J) were provided by the RIKEN BRC through the National BioResource Project of the MEXT/AMED, Japan.
Wild animals	This study did not involve wild animals.
Reporting on sex	Both male and female mice were used at 8-10 weeks old with age- and sex-matched controls.
Field-collected samples	No samples collected from the field were used.
Ethics oversight	All animal experiments were approved by the Institutional Animal Care and Use Committee of Hokkaido University (permission number: 19-0147)

Note that full information on the approval of the study protocol must also be provided in the manuscript.

Flow Cytometry

Plots

Confirm that:

☒ The axis labels state the marker and fluorochrome used (e.g. CD4-FITC).

☒ The axis scales are clearly visible. Include numbers along axes only for bottom left plot of group (a 'group' is an analysis of identical markers).

☒ All plots are contour plots with outliers or pseudocolor plots.

☒ A numerical value for number of cells or percentage (with statistics) is provided.

Methodology

Sample preparation	For T cells: Naive CD4+ T cells were isolated from the murine spleen by magnetic cell sorting with the naive CD4+ T Cell Isolation kit (Miltenyi Biotec). Approximately 0.3 million naive CD4+ T cells were plated into 48-well-plate pre-coated with goat anti-hamster IgG (MP Biomedicals) and stimulated for 2–3 days with anti-CD3 (0.25 μg/mL, clone 145-2C11, Biolegend, 100340) and anti-CD28 (0.5 μg/mL, clone 37.51, Biolegend, 102116) antibodies. For each T cell differentiation, subset-specific antibodies and cytokines were further supplemented. After fixation and permeabilisation, the cells were stained with antibodies.<br>For bone-marrow-derived macrophage: Bone marrow cells were harvested from the femur and tibia of C57BL/6J mice and differentiated in the presence of M-CSF (20 ng/mL, R&D Systems, 416-ML-010) in RPMI 1640 medium containing 10% FBS, 0.1% 2-mercaptoethanol, and penicillin-streptomycin at 37 °C under 5% CO2 for 8 days. On day 8, the bone-marrow-derived macrophage were washed and stimulated with lipopolysaccharide (LPS, 100 ng/mL, Sigma-Aldrich, L2880) for 6 h.
Instrument	FACS Aria III (BD) was used for multi-parameter analysis.
Software	FlowJo v10.6.1 (BD) was used for data analysis.
Cell population abundance	Purity > 95% as assessed by post-sort measurements of samples.
Gating strategy	In brief, events were initially gated by FSC and SSC (to exclude doublets), then by exclusion of dead events using zombie aqua-BV510. Subsequent gating depended on the population of interest and is outlined in the supplementary Fig. 1a and 2e.

☒ Tick this box to confirm that a figure exemplifying the gating strategy is provided in the Supplementary Information.
